# The Application of High-Performance Silver Nanowire and Metal Oxide Composite Electrodes as Window Electrodes in Electroluminescent Devices

**DOI:** 10.3390/mi17010141

**Published:** 2026-01-22

**Authors:** Xingzhen Yan, Ziyao Niu, Mengying Lyu, Yanjie Wang, Fan Yang, Chao Wang, Yaodan Chi, Xiaotian Yang

**Affiliations:** 1 Key Laboratory of Architectural Cold Climate Energy Management, Ministry of Education, Jilin Jianzhu University, 5088 Xincheng Street, Changchun 130118, China; 13343197732@163.com (Z.N.); 17303761673@163.com (M.L.); wangyanjie@jlju.edu.cn (Y.W.); ctpnxn@163.com (F.Y.); wangchao@jlju.edu.cn (C.W.); chiyaodan@jlju.edu.cn (Y.C.); 2 Jilin Provincial Key Laboratory of Architectural Electricity & Comprehensive Energy Saving, Jilin Jianzhu University, Changchun 130118, China

**Keywords:** metal nanowire, transparent conductive film, luminescence intensity, optoelectronic device

## Abstract

In this paper, composite structures were fabricated by incorporating silver nanowires (AgNWs) with various metal oxides via the sol–gel method. This approach enhanced the electrical performance of AgNW-based transparent electrodes while simultaneously improving their stability under damp heat conditions and modifying the local medium environment surrounding the AgNW meshes. The randomly distributed AgNW meshes fabricated via drop-coating were treated with plasma to remove surface organic residues and reduce the inter-nanowire contact resistance. Subsequently, a zinc oxide (ZnO) coating was applied to further decrease the sheet resistance (R_sheet_) value. The pristine AgNW mesh exhibits an R_sheet_ of 17.4 ohm/sq and an optical transmittance of 93.06% at a wavelength of 550 nm. After treatment, the composite structure achieves a reduced R_sheet_ of 8.7 ohm/sq while maintaining a high optical transmittance of 92.20%. The use of AgNW meshes as window electrodes enhances electron injection efficiency and facilitates the coupling mechanism between localized surface plasmon resonances and excitons. Compared with conventional ITO transparent electrodes, the incorporation of the AgNW mesh leads to a 17-fold enhancement in ZnO emission intensity under identical injection current conditions. Moreover, the unique scattering characteristics of the AgNW and metal oxide composite structure effectively reduce photon reflection at the device interface, thereby broadening the angular distribution of emitted light in electroluminescent devices.

## 1. Introduction

Transparent electrodes are essential components in wearable devices, display panels, and solar cells [1,2,3,4]. With the advancement of flexible electronics, conventional transparent metal oxide electrodes have become increasingly inadequate to meet the performance requirements of modern devices [5,6]. Among various low-dimensional materials suitable for transparent electrode fabrication, randomly distributed silver nanowire (AgNW) meshes have emerged as one of the most promising candidates due to their superior electrical conductivity, high optical transmittance across a broad spectrum, and excellent mechanical flexibility [7,8,9]. The Cui and Peumans group demonstrated that a randomly distributed AgNW mesh fabricated through a simple solution-based process exhibits superior optoelectronic performance compared to conventional ITO thin films and carbon nanotube grid structures. Notably, the optoelectronic properties in the high-transmittance regime closely approach the theoretical predictions for an ideal metallic silver grid [10].

Although AgNWs have demonstrated notable advantages in optoelectronic performance as transparent electrodes, solution-processed AgNW meshes continue to face challenges associated with high initial sheet resistance (R_sheet_) and limited environmental stability [11,12]. To address the aforementioned issues associated with AgNW meshes, researchers have focused on constructing composite structures by integrating AgNWs with organic polymers [13,14], graphene [15], and inorganic metal oxides [16,17,18] to mitigate high surface roughness and enhance environmental stability under ambient conditions. Meanwhile, various welding techniques have been developed to reduce the contact resistance at the junctions between AgNWs [19,20]. By utilizing the environmental stability of metal oxides, solution-processed composite electrodes consisting of AgNW meshes and metal oxides have been developed to enhance electron injection efficiency in optoelectronic devices. Meanwhile, metal oxides enable tunability of the plasma resonance peaks in AgNWs. The impact of enhanced coupling between localized surface plasmons in AgNWs and ZnO excitons on optical emission efficiency merits further investigation.

In this study, the drop-coating method was employed to fabricate randomly distributed AgNW electrodes with a high aspect ratio. To remove the polyvinylpyrrolidone (PVP) layer adhering to the AgNW surface during synthesis, nitrogen plasma treatment was proposed as a surface modification strategy. This non-contact method effectively reduces residual organic contaminants on the pristine AgNW surface and lowers the R_sheet_ of the resulting electrode. The environmental stability of AgNW meshes was significantly enhanced through integration with metal oxides. The electrical properties of the composite films remained stable under high-temperature and high-humidity environmental conditions. The application of AgNW and metal oxide composite electrodes in electroluminescent devices has demonstrated that the AgNW mesh not only enhances electron injection efficiency but also significantly improves the light output efficiency of the device through localized surface plasmon resonance with ZnO excitons. Compared with conventional ITO electrodes, the unique scattering properties of the nanostructures in the composite film result in a broader angular distribution of emitted light.

## 2. Experimental Procedures

### 2.1. Materials

Commercial AgNWs with diameters of approximately 50 nm and 30 nm and an average length of 100–200 μm were dispersed in ethanol to prepare a homogeneous solution at a concentration of 61.5 μg/mL. For AgNWs with a diameter of 50 nm, five samples with deposition concentrations of 3.690 μg/cm^2^, 4.305 μg/cm^2^, 4.920 μg/cm^2^, 5.535 μg/cm^2^, and 6.150 μg/cm^2^ were fabricated by drop-coating 40 μL of AgNWs solution onto 2 × 2 cm glass substrates at 1 min intervals for 6, 7, 8, 9, and 10 cycles, respectively. For AgNWs with a diameter of 30 nm, five samples with deposition concentrations of 5.535 μg/cm^2^, 6.765 μg/cm^2^, 7.995 μg/cm^2^, 9.225 μg/cm^2^, and 10.455 μg/cm^2^ were fabricated by drop-coating 40 μL of AgNWs solution onto 2 × 2 cm glass substrates at 1 min intervals for 9, 11, 13, 15, and 17 cycles, respectively. A 0.1 M ZnO precursor solution was prepared by dissolving 0.11 g of zinc acetate dihydrate (Zn(CH_3_COO)_2_·2H_2_O) in 5 mL of anhydrous ethanol, followed by the addition of 0.031 mL of ethanolamine as a stabilizing agent and stirring the mixture at 60 °C for 2 h. Aluminum-doped zinc oxide (AZO), indium-doped zinc oxide (IZO), and gallium-doped zinc oxide (GZO) precursor solutions with a concentration of 0.1 M were prepared separately by dissolving 0.019 g of aluminum nitrate nonahydrate (Al(NO_3_)_3_·9H_2_O) and 0.101 g of zinc acetate dihydrate, 0.015 g of indium nitrate hydrate (In(NO_3_)_3_·xH_2_O) and 0.099 g of zinc acetate dihydrate, or 0.013 g of gallium nitrate hydrate (Ga(NO_3_)_3_·xH_2_O) and 0.100 g of zinc acetate dihydrate, respectively, in 5 mL of anhydrous ethanol, following the same procedure as described above. All the precursor materials were purchased from Aladdin Technology Co., Ltd., Shanghai, China.

### 2.2. Preparation of Composite Films

**Figure 1:**
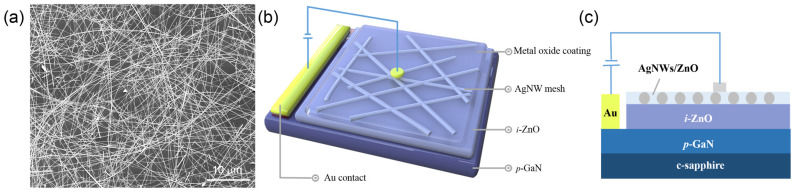
(**a**) SEM image of a randomly distributed AgNW mesh structure. (**b**) Schematic illustration and (**c**) side-view illustration of the electroluminescent device structure incorporating an AgNW mesh as the transparent window electrode.

ZnO films with a thickness of approximately 50 nm were deposited by magnetron sputtering onto commercial p-type GaN:Mg/c-sapphire substrates. The deposition process was conducted in a mixed atmosphere consisting of 80% argon and 20% oxygen at a growth pressure of 12 mTorr. The deposition was performed for 35 min at a sputtering power of 100 W. The ZnO/p-GaN substrate was then subjected to thermal annealing at 300 °C for 1 h in an oxygen atmosphere. Subsequently, the annealed ZnO/p-GaN substrate was subjected to nitrogen plasma treatment at a power of 112 W for 3 min to improve the wettability of the ZnO surface and promote the uniform distribution of AgNWs. Then, AgNWs with a diameter of 30 nm and a deposition concentration of 9.225 μg/cm^2^ were deposited onto the substrate by drop-coating to form a randomly distributed AgNW mesh structure (see Figure 1a). Following nitrogen plasma treatment for 60 s, the filtered metal oxide precursor solution was spin-coated onto the AgNW mesh surface at 4000 rpm for 30 s. This plasma treatment facilitated the spin-coating of metal oxides onto AgNW meshes and effectively reduced residual organic contaminants on the nanowire surfaces. The resulting film was then transferred to a hot plate maintained at 80 °C and cured for 10 min. The ITO films were prepared by magnetron sputtering for 40 min at a pressure of 25 mTorr in an argon atmosphere, at a sputtering power of 70 W, and a substrate temperature of 200 °C, and were used as a comparative counterpart to the AgNW/metal oxide composite structure. The target material composition consisted of In_2_O_3_ and SnO_2_ in a molar ratio of 9:1. Following annealing in a nitrogen atmosphere at 250 °C for 1 h, the ITO film with a thickness of approximately 70 nm exhibited a R_sheet_ of 74.6 ohm/sq and an optical transmittance of 91.06% at 550 nm. The corresponding device architecture is schematically illustrated in Figure 1b,c.

### 2.3. Characterization

The R_sheet_ of the transparent electrodes was measured by a four-probe tester (FT-341, Rooko, Ningbo, China). Transmittance spectra were measured using a UV/Vis spectrophotometer (UV-2600, Shimadzu, Fukuoka, Japan). The intrinsic ZnO layer and ITO electrode were deposited using a physical vapor deposition system (PVD75, Kurt J. Lesker, Pittsburgh, PA, USA). The microstructures of the AgNW meshes and composite films were characterized using a field emission scanning electron microscope (SEM, JSM-7610F, JEOL, Tokyo, Japan). Electroluminescence measurements were conducted using a spectrometer (QEPro, OceanOptics, Dunedin, FL, USA). Photoluminescence measurements were performed using a spectroscopic detection system (iHR550, Horiba, Paris, France).

## 3. Results and Discussion

**Figure 2:**
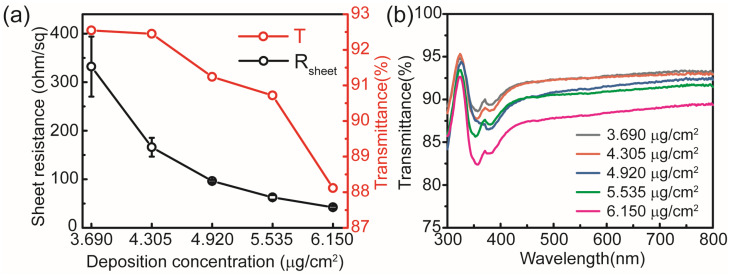
(**a**) R_sheet_ and optical transmittance at 550 nm of AgNW meshes with varying deposition concentrations. (**b**) Transmission spectra of the AgNW mesh electrodes.

AgNW meshes have emerged as a promising candidate for transparent electrode applications due to their excellent photoelectric properties and broad-spectrum optical transparency. Five samples with varying AgNW concentrations were fabricated on glass substrates by controlling the number of deposition cycles, using individual AgNWs with an average diameter of 50 nm and a length of 100–200 μm. The R_sheet_ and transmittance of each sample were measured using the four-point probe method and a UV/Vis spectrophotometer, respectively, as shown in Figure 2. Figure 2a exhibits the R_sheet_ and transmittance at 550 nm for AgNW meshes with different deposition concentrations. Figure 2b shows the transmission spectra of the AgNW mesh electrodes. The AgNW electrodes exhibit good full-spectrum transmittance when used as window electrodes. Since light transmits through the interstitial regions of the AgNW mesh, an increase in deposition concentration results in decreased optical transmittance and a lower R_sheet_, as shown in Table 1.

**Figure 3:**
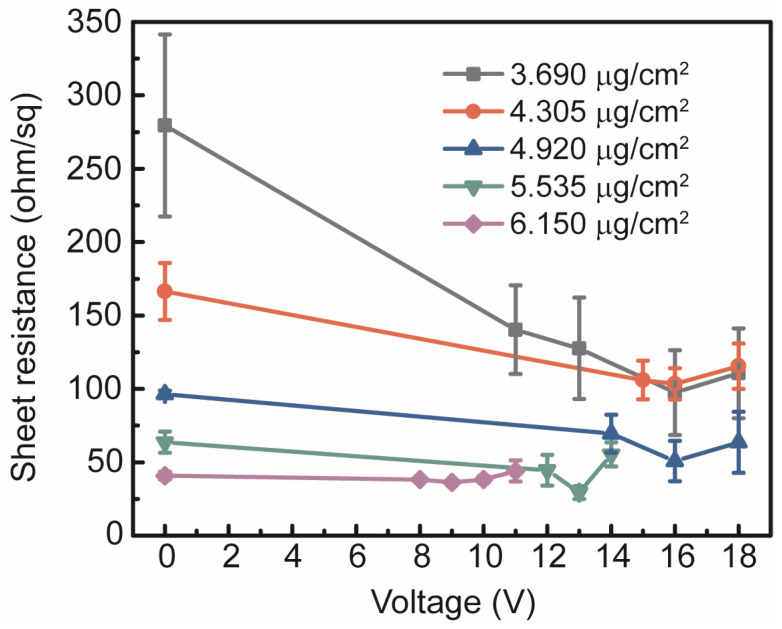
Variation in R_sheet_ of AgNW meshes with different deposition concentrations as a function of applied electro-sintering voltage.

The AgNW meshes fabricated via the drop-coating method primarily rely on their own weight to form random overlaps, leading to loosely connected junctions that result in poor electrical contact at intersection points and consequently a high R_sheet_. Hence, a further reduction in the R_sheet_ in the as-prepared AgNW meshes is required. Electro-sintering has been proposed as a method to address these limitations. Localized Joule heating is generated at overlapping junctions by applying a voltage across the film using an adjustable DC power supply. Localized melting and welding significantly reduce the resistance and enhance the conductive pathway. As shown in Figure 3, the R_sheet_ values of the pristine AgNW meshes with five different deposition concentrations decreased upon the application of the corresponding electro-sintering voltages. The R_sheet_ of the AgNW mesh fabricated with a deposition concentration of 5.535 μg/cm^2^ was effectively reduced from 63.6 to 29.3 ohm/sq after electro-sintering at 13 V for 2 min. The effective sintering voltage decreased with increasing AgNW concentration. However, a sharp increase in R_sheet_ was observed for each sample group upon application of excessive voltage. When a sintering voltage of 14 V was applied across the AgNW mesh, the R_sheet_ increased sharply to 55.3 ohm/sq.

**Figure 4:**
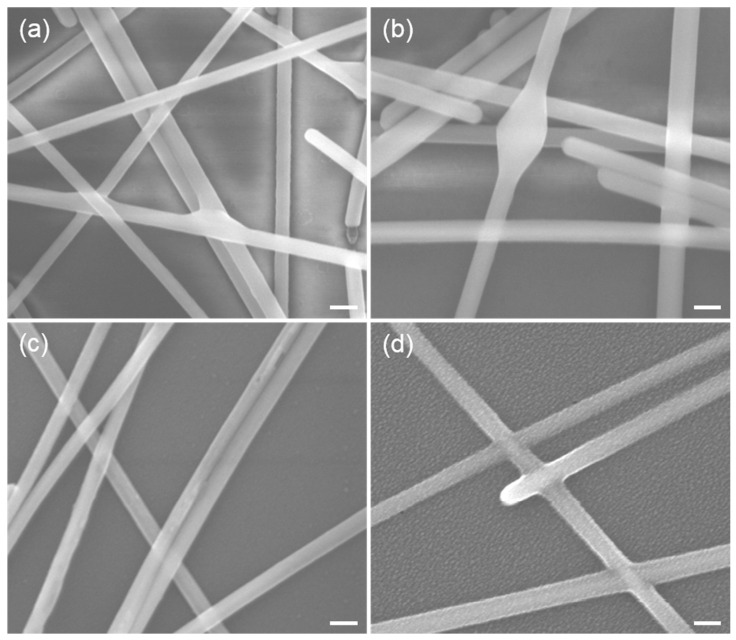
Scanning electron microscope images of AgNW mesh electrodes after electro-sintering (**a**), over-electro-sintering (**b**), nitrogen plasma treatment (**c**), and composite formation with metal oxides (**d**). The scale bar is 100 nm.

The SEM images reveal the surface morphological changes responsible for the R_sheet_ variations in the AgNW mesh with a deposition concentration of 5.535 μg/cm^2^ under different sintering voltages. As shown in Figure 4a, the application of a 13 V sintering voltage induced the formation of welded structures at the AgNW junctions, thereby significantly reducing the R_sheet_ of the AgNW mesh. In contrast, further voltage escalation led to localized melting of the AgNWs at their overlapping junctions, resulting in a sharp increase in R_sheet_ (see Figure 4b). The AgNW meshes were subjected to an annealing treatment at 170 °C for 10 min to evaluate whether thermal annealing could further enhance junction fusion. The R_sheet_ of the AgNW mesh decreased only by approximately 3 ohm/sq. This result indicates that the electro-sintering treatment has already established effective welding at the overlapping regions of the AgNWs.

The AgNWs are held together solely through physical contact and exhibit poor adhesion to the substrate, which limits their suitability as electrodes in electronic devices. To address this issue and enhance environmental stability, a metal oxide coating layer was applied to protect the AgNWs from oxidation. Nitrogen plasma treatment was applied to the prepared AgNW mesh for 60 s to improve surface wettability. Meanwhile, it was found that plasma treatment can remove organic contaminants, such as polyvinylpyrrolidone (PVP), generated during the synthesis of AgNWs and enhance interconnectivity among the AgNWs. As shown in Figure 4c, the surface of the AgNWs becomes roughened under plasma treatment. The R_sheet_ of the AgNW mesh was reduced to 20 ohm/sq after plasma treatment. As shown in Figure 4d, a composite electrode structure consisting of AgNW meshes and ZnO was fabricated by spin-coating a ZnO precursor solution onto the AgNWs. The AgNW meshes used for comparison were fabricated under identical environmental and concentration conditions to ensure experimental comparability. The R_sheet_ of the composite film was 15.6 ohm/sq with a transmittance of 88.87% at 550 nm.

**Figure 5:**
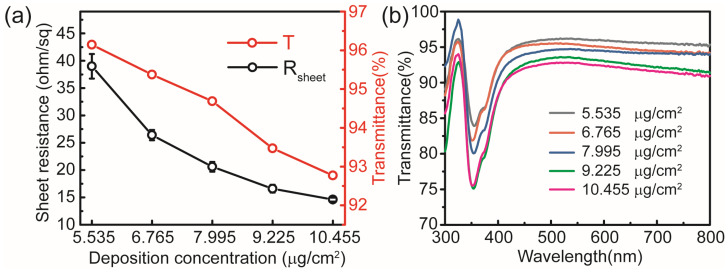
(**a**) R_sheet_ and optical transmittance at 550 nm of AgNW meshes (30 nm in diameter) with varying deposition concentrations. (**b**) Transmission spectra of the AgNW mesh electrodes.

The above experiments demonstrate that AgNWs with a diameter of 50 nm still exhibit limitations in optical transmittance. Therefore, AgNWs with a smaller diameter of 30 nm and a length of 100–200 μm were selected to fabricate an AgNW-based transparent electrode with a R_sheet_ below 10 ohm/sq and an optical transmittance exceeding 90% at 550 nm. The R_sheet_ and optical transmittance of the AgNW meshes with varying deposition concentrations are presented in Figure 5. Considering the R_sheet_ and optical transmittance of the AgNW meshes comprehensively, the sample with a deposition concentration of 9.225 μg/cm^2^ was selected for subsequent performance optimization and as a window electrode in electroluminescent devices. In contrast, the AgNW mesh with a diameter of 30 nm exhibits enhanced performance relative to its 50 nm counterpart in both initial R_sheet_ and optical transmittance. For the AgNW samples with a diameter of 30 nm, direct compositing with metal oxides was conducted following plasma treatment. The R_sheet_ of the pristine AgNW mesh electrode decreased from approximately 17 to 9.3 ohm/sq following nitrogen plasma treatment. Subsequently, 40 μL of 0.1 M solutions of various metal oxide precursors were spin-coated onto the AgNW meshes, followed by curing at 80 °C. The resulting changes in R_sheet_ and transmittance of the various AgNW/metal oxide composite films are summarized in Table 2.

**Figure 6:**
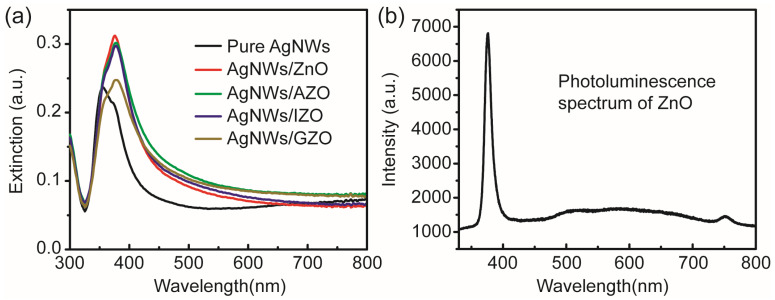
(**a**) Extinction spectra of pure AgNWs and various composite films. (**b**) Photoluminescence spectrum of the ZnO luminescent layer.

The AgNW mesh structure employed as an electrode can significantly enhance electron transport properties in optoelectronic devices and also serves as a localized surface plasmon source to improve the exciton recombination efficiency in electroluminescent devices. The effective combination of AgNWs with ZnO-based light-emitting devices can significantly enhance the luminescence efficiency of ZnO excitons. As shown in Figure 6a, the surface plasmon resonance peak position of AgNWs and its shift upon incorporation of composite metal oxides were investigated by measuring the extinction spectra of the AgNWs and the corresponding composite films. The plasmon resonance peak position exhibited a significant redshift after AgNWs were combined with metal oxides. However, the surface plasmon resonance peak positions of the AgNW-based composite structures are very similar due to the relatively thin layers of different types of metal oxide coatings. In Figure 6b, the photoluminescence spectra of the ZnO luminescent layer fabricated by magnetron sputtering were measured to characterize the band-edge emission peak positions of ZnO. A 325 nm He–Cd laser was used as the excitation source to investigate the intrinsic luminescence properties of ZnO [21,22]. The surface plasmon resonance peak of AgNWs/ZnO is closer to the band-edge emission peak of intrinsic ZnO (~376 nm). The resonant coupling between the localized surface plasmons of AgNWs and ZnO excitons enhances the luminescence intensity of ZnO. Considering the material homogeneity with the ZnO luminescent layer, a composite structure composed of AgNWs and ZnO coatings was selected as the window electrode for integration into the i-ZnO/p-GaN light-emitting device. The corresponding structural schematic is shown in Figure 1b,c.

**Figure 7:**
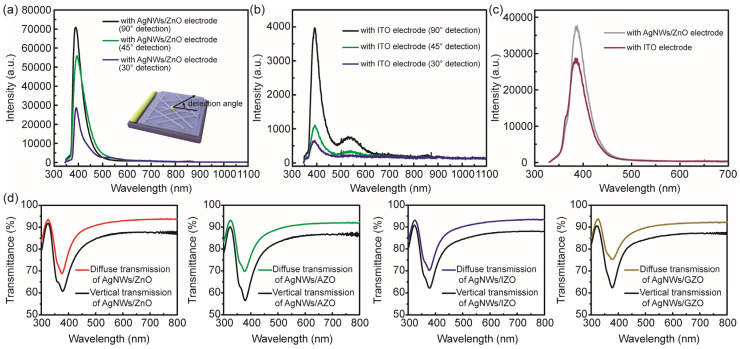
Electroluminescence spectra of AgNWs/ZnO composite structures (**a**) and ITO (**b**) as electrodes in i-ZnO/p-GaN light-emitting devices measured at different detection angles. (**c**) Photoluminescence spectra of the AgNWs/ZnO and ITO as electrodes in devices. (**d**) Diffuse and vertical transmission spectra of the AgNWs/ZnO, AgNWs/AZO, AgNWs/IZO, and AgNWs/GZO composite films.

As shown in Figure 7, the electroluminescence spectra of the i-ZnO/p-GaN light-emitting device using AgNWs/ZnO composite structures and magnetron sputtered ITO as electrodes are presented as a function of detection angle under a constant injection of 10 mA (corresponding to a current density of 2598.4 mA/cm^2^). The same optical setup was used to measure the electroluminescence performance of the AgNW/ZnO composite structure and ITO as window electrodes in light-emitting devices. At a detection angle of 90°, the luminescence intensity of the device using an AgNWs/ZnO composite electrode was approximately 17 times that of the same device fabricated with an ITO conductive film. The electroluminescence intensities of devices using AgNWs/ZnO composite films and ITO as window electrodes were compared at different detection angles, as presented in Table 3. To further support the plasmonic enhancement interpretation, we present the photoluminescence spectra of i-ZnO/p-GaN light-emitting devices fabricated with AgNWs/ZnO and ITO electrodes in Figure 7c. A 325 nm He–Cd laser was employed as the excitation source. The localized surface plasmon resonance peak of AgNWs is close to the emission peak of ZnO, which enhances the light extraction efficiency. The R_sheet_ of the AgNWs/ZnO composite electrode was reduced by about 8 times compared to that of ITO. The remaining enhancement in electroluminescence intensity was attributed to plasmon-exciton coupling between AgNWs and ZnO excitons, as well as to the improved current spreading capability of the AgNW mesh. It is noteworthy that the luminescence intensity achieved with the AgNWs/ZnO composite structure as the window electrode is significantly higher than that obtained with the ITO electrode as the detection angle gradually decreases. This can be attributed to the modification of metal oxide particles on the surface of AgNW meshes, which enhances the light scattering characteristics of the composite nanostructure, thereby enabling the light-emitting device to maintain a more stable emission intensity under small-angle detection conditions.

**Figure 8:**
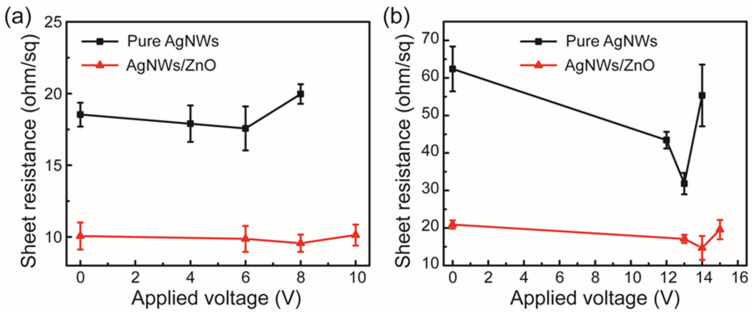
Comparison of the electrical stability between the AgNWs/ZnO composite structure and the pure AgNW meshes with nanowire diameters of 30 nm (**a**) and 50 nm (**b**).

**Figure 9:**
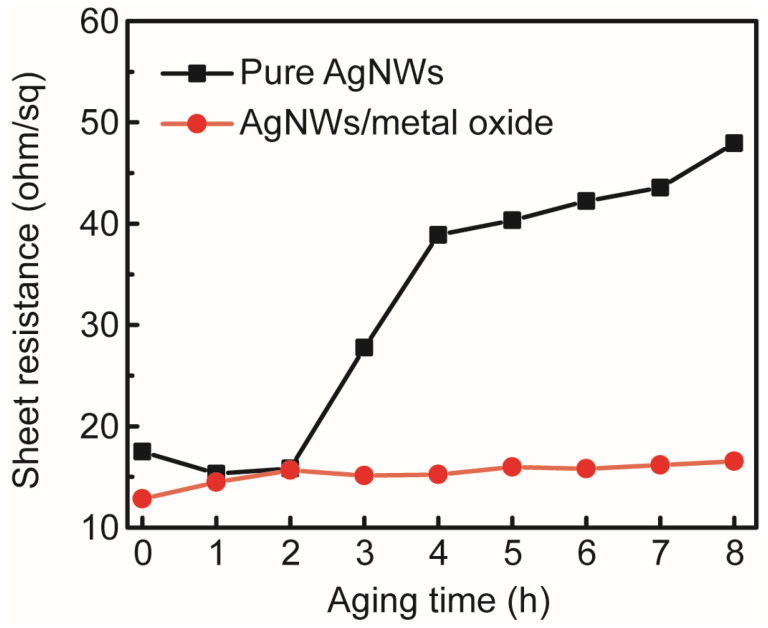
Temporal evolution of the R_sheet_ in pure AgNW meshes and AgNW/metal oxide composite structures under high-temperature and high-humidity conditions.

To verify this, the vertical optical transmission and diffuse transmission spectra of AgNWs combined with various metal oxides were characterized in Figure 7d. The diffuse transmittance (measured using the integrating sphere module of the UV-2600) was more than 5% higher than the corresponding vertical transmittance (measured using the vertical module of the UV-2600) for all composite structures, as shown in Table 4. The AgNW mesh and metal oxide composite structure employed as a window electrode exhibited full-spectrum transmittance and wavelength-independent light scattering characteristics. The AgNW-based transparent electrodes exhibited significant advantages in electroluminescence intensity and angular distribution of emitted light when employed in light-emitting devices. The surface roughness of the pristine AgNW mesh was approximately 6.02 nm. Spin-coating of ZnO via solution processing reduced the surface roughness to 5.52 nm, demonstrating effective filling of AgNW meshes through solution processing. In addition, the AgNW mesh coated with metal oxides exhibited enhanced voltage resistance compared to the pure AgNW mesh when a voltage was applied across the film. This improvement was consistently observed in AgNW meshes with a diameter of 50 nm (see Figure 8). Meanwhile, the metal oxide coating could also enhance the environmental stability of the AgNW mesh electrode. As shown in Figure 9, the R_sheet_ of the composite structure remained relatively stable compared to that of pure AgNW meshes under high-temperature and high-humidity conditions (~85 °C and 85% RH).

## 4. Conclusions

In conclusion, we propose a composite structure consisting of an AgNW mesh and a sol–gel-derived metal oxide as a window electrode for electroluminescent devices. The R_sheet_ of the pristine AgNW mesh electrode decreased from approximately 17 to 9.3 ohm/sq after nitrogen plasma treatment. The plasma treatment can effectively remove the organic polymer layer adhering to the surface of AgNWs during the synthesis process. It significantly reduced the R_sheet_ of the AgNW meshes by increasing the contact area at the overlaps within the mesh. The prepared AgNW mesh was coated with ZnO, resulting in a reduction in R_sheet_ to 8.7 ohm/sq and an optical transmittance of 92.20% at a wavelength of 550 nm. The surface plasmon resonance peak of AgNWs was shifted to align with the band-edge emission peak of intrinsic ZnO through the construction of composite structures composed of AgNWs and metal oxides. The application of the composite films as window electrodes in electroluminescent devices not only enhanced electron injection efficiency but also improved light emission efficiency through the coupling mechanism between localized surface plasmons in AgNWs and ZnO excitons. Compared with traditional ITO transparent electrodes, the incorporation of AgNWs and metal oxides enhanced the ZnO emission intensity by more than 17-fold under identical injection current conditions. Meanwhile, the composite electrodes exhibited significant advantages in the angular distribution of emitted light when employed in electroluminescent devices.

## Figures and Tables

**Table 1:** Comparison of R_sheet_ and optical transmittance for pristine AgNW mesh electrodes.

Sample	Sheet Resistance (ohm/sq)	Transmittance (%) at 550 nm
3.690 μg/cm^2^	332.1	92.55
4.305 μg/cm^2^	165.9	92.45
4.920 μg/cm^2^	96.2	91.24
5.535 μg/cm^2^	62.6	90.72
6.150 μg/cm^2^	42.4	88.12

**Table 2:** Comparison of the R_sheet_ and optical transmittance of AgNW/metal oxide composite films.

Sample	Sheet Resistance (ohm/sq)	The Changes in Transmittance (%) at 550 nm Before and After Compounding
AgNWs/ZnO	8.7	93.06→92.20%
AgNWs/AZO	9.4	92.40→90.63%
AgNWs/IZO	9.5	92.82→91.52%
AgNWs/GZO	9.7	92.63→90.89%

**Table 3:** Comparison of the luminescence intensity between AgNW/ZnO composite films and ITO as window electrodes under identical current density conditions at various detection angles.

Sample	Current Density (mA/cm^2^)	Luminescence Intensity at 90° (a.u.)	Luminescence Intensity at 60° (a.u.)	Luminescence Intensity at 30° (a.u.)
AgNWs/ZnO	2598.4	70,920.9	55,957.0	28,602.3
ITO	2598.4	3972.0	1079.5	660.6

**Table 4:** Comparison of diffuse transmittance and vertical transmittance in AgNWs and metal oxide composites.

Sample	Diffuse Transmittance (%) at 550 nm	Vertical Transmittance (%) at 550 nm	Difference Value
AgNWs/ZnO	92.20%	86.64%	5.56%
AgNWs/AZO	90.63%	85.24%	5.39%
AgNWs/IZO	91.52%	86.08%	5.44%
AgNWs/GZO	90.89%	85.79%	5.10%

## Data Availability

The original contributions presented in this study are included in the article. Further inquiries can be directed to the corresponding authors.
